# Multi-Locus GWAS Mapping and Candidate Gene Analysis of Anticancer Peptide Lunasin in Soybean (*Glycine max* L. Merr.)

**DOI:** 10.3390/plants14142169

**Published:** 2025-07-14

**Authors:** Rikki Locklear, Jennifer Kusumah, Layla Rashad, Felecia Lugaro, Sonia Viera, Nathan Kipyego, Faith Kipkosgei, Daisy Jerop, Shirley Jacquet, My Abdelmajid Kassem, Jiazheng Yuan, Elvira de Mejia, Rouf Mian

**Affiliations:** 1 Department of Biological and Forensic Sciences, Fayetteville State University, Fayetteville, NC 20348, USA; rlocklear2@broncos.uncfsu.edu (R.L.); laylar00@outlook.com (L.R.); flugaro@broncos.uncfsu.edu (F.L.); sonia_viera@outlook.com (S.V.); nkipyego@broncos.uncfsu.edu (N.K.); fkipkosger@broncos.uncfsu.edu (F.K.); djerop@broncos.uncfsu.edu (D.J.); sjacquet@broncos.uncfsu.edu (S.J.); mkassem@uncfsu.edu (M.A.K.); 2 Department of Food Science & Human Nutrition, University of Illinois at Urbana-Champaign, Urbana, IL 61801, USA; kusumah2@illinois.edu; 3 Department of Biological Sciences, Old Dominion University, Norfolk, VA 23529, USA; 4 Soybean and Nitrogen Fixation Research Unit, The United States Department of Agriculture—Agricultural Research Service, Raleigh, NC 27607, USA

**Keywords:** soybean, lunasin, anticancer, mult-locus GWAS, QTN, candidate genes

## Abstract

Soybean (*Glycine max*) peptide lunasin exhibits significant cancer-preventive, antioxidant, and hypocholesterolemic effects. This study aimed to identify quantitative trait nucleotides (QTNs) associated with lunasin content and to annotate the candidate genes in the soybean genome. The mapping panel of 144 accessions was gathered from the USDA Soybean Germplasm Collection, encompassing diverse geographical origins and genetic backgrounds, and was genotyped using SoySNP50K iSelect Beadchips. The lunasin content in soybean seeds was measured using the enzyme-linked immunosorbent assay (ELISA) method, with lipid-adjusted soybean flour prepared from seeds obtained from the Germplasm Resource Information Network (GRIN) of USDA-ARS in 2003 and from North Carolina in 2021, respectively. QTNs significantly related to lunasin content in soybean seeds were detected on 15 chromosomes, with LOD scores greater than 3.0, explaining various phenotypic variations identified using the R package mrMLM (v4.0). Significant QTNs on chromosomes 3, 13, 16, 18, and 20 were consistently identified across multiple models as being significantly associated with soybean lunasin content, based on assessment data from two years. Twenty-nine candidate genes were found, with 12 identified in seeds from 2003 and 17 from 2021. Our study is an important effort to understand the genetic basis and functional genes for lunasin production in soybean seeds.

## 1. Introduction

Improving seed composition and quality, including protein, oil, fatty acid, and amino acid content, is an important aim of soybean farmers and breeders. Soybean (*Glycine max*) consumption has been associated with beneficial effects in improving human health such as reducing obesity, cardiovascular disease, immune disorders, and certain types of cancers [1,2]. Diets rich in soybean products are associated with lower colon, breast, and prostate cancer mortalities, suggesting that carcinogenesis prevention may be derived from soybean components [3]. The therapeutic function of soybean has also been recognized in soy bioactive peptides, both from soy protein in seeds and peptides derived through gastrointestinal digestion [4]. Lunasin has shown remarkable cancer-preventive, antioxidant, and hypocholesterolemic effects in animal and in vitro trials [5,6,7,8,9,10,11,12,13]. Since it was isolated from soybean seeds in 1981 during the screening of protease inhibitors in Japan, peptide lunasin has been considered one of the most promising potential anticancer phytochemicals [9]. Besides its anticancer activity, lunasin plays a vital role in the regulation of cholesterol biosynthesis in the body with its inherent antioxidative and anti-inflammatory effects [14]. Lunasin possesses high tissue affinity, specificity, and efficiency in promoting human health, and has been associated with antihypertention, antiobesity, and anticancer properties [15]. These various biological functions of lunasin have been clearly demonstrated in both in vitro and in vivo assessments [9,10,16]. Lunasin displays the ability to regulate the cell cycle through inducing cell apoptosis, decreasing the gene expression of cyclin, inhibiting oncogene expression, and reducing the mutation rate caused by carcinogens [8,16,17]. A study suggested that mice receiving lunasin treatment showed significantly reduced pulmonary colonization after the injection of highly metastatic melanoma cells compared to the control group [18]. Lunasin inhibits the growth of murine LLC cells and murine B16-F0 melanoma cells in vitro and in wild-type C57BL/6 mice [19]. Lunasin can help prevent metastasis and patient relapses in melanoma by reducing the invasive potential of cancer initiation cells (CICs), shown both in vitro and in vivo in a mouse experimental metastasis model, and therefore, lunasin has been considered an exceptional anticancer agent for patients that have developed resistance to current chemotherapies [18].

Overall, tremendous progress has been made in understanding the anticancer bioactive functions of lunasin. However, the genetic basis and inheritance of lunasin have not been fully addressed, and QTL mapping and candidate genes that are associated with lunasin production in soybeans have not been defined genetically to the best of our knowledge. More studies are required to elucidate the genetic mechanisms of production for lunasin in soybean seeds to bridge the gap between advanced clinical therapeutic success and the lack of genetic information on lunasin inheritance. de Mejia et al. [20] quantified lunasin concentrations in 144 selected, diverse soybean accessions (*Glycine max*) from the USDA Soybean Germplasm Collection (Urbana, Illinois), indicating that the lunasin concentrations within soybean accessions vary greatly. These 144 accessions from USDA Soybean Germplasm Collection, including exotic, ancestral, and modern accessions, were quantified in soybean defatted flour using an enzyme-linked immunosorbent assay (ELISA) method [20]. In this study, lunasin concentrations in the flour of soybean lines varied greatly and significantly, ranging from 0.1 to 1.3 g/100 g in exotic lines, 3.6 to 10.1 g/100 g in ancestral lines, and 3.3 to 9.5 g/100 g in modern lines. Notably, the maximum differences in lunasin content exceeded 100% among soybean lines grown under the same environmental conditions. These findings suggest that genetic background plays a major role in determining lunasin accumulation, even when environmental factors are held constant. The average lunasin concentration in 23 major ancestral lines of U.S. cultivars appears to be like that of 16 modern cultivars; the ancestral and exotic accessions show the highest value of lunasin concentration. Interestingly, accessions with high concentrations of isoflavone-enriched products contain low or no lunasin. Moreover, the variation in lunasin concentration is correlated with the accession’s geological origin but less likely related to soybean growth maturity based on pioneer research [20]. Abiotic stress, such as in salt-treated soybean seeds, has been found to be able to induce lunasin accumulation at the highest level six hours after imbibition [21]. The phenotypic variation in lunasin content in soybean seeds of diverse soybean lines has positioned lunasin as an ideal candidate trait for the genetic improvement of luansin concentrations in soybean plants.

The genome-wide association study (GWAS) is a powerful alternative approach to the traditional biparental mapping approach for mapping the target QTL of complex traits at sequence resolution by accounting for the population structure and historical recombination events in mapping panels as co-variances in the mathematic model [22,23,24,25]. It has been widely used to investigate the various traits of plants, such as maize [26], soybean [23,27], barley [28,29], *Arabidopsis thaliana* [30], and sorghum [31]. The GWAS is one of the most powerful methods. The GWAS has been widely used to define quantitative trait loci because it will directly detect a genetic association between single-nucleotide polymorphism (SNP) markers and traits in the mapping panels of landraces and advanced breeding populations based on linkage disequilibrium (LD) information [23,32]. The mixed linear model (MLM) is commonly implemented for GWAS analysis, which combines historical recombination events, a greater number of alleles, and broader genetic variation as co-variances to dissect the genetic merits of complex traits [33,34,35]. However, single-locus models such as the CMLM [35] and the ECMLM [35] are one-dimensional genome scans, which need corrections for multiple tests. The Bonferroni correction is often integrated for multiple tests, and nevertheless, this stringent correction has some deficiencies in the identification of QTLs with small effects, particularly in field experiments on crop genetics [36]. Recently, Wang et al. [26] proposed a multi-locus random-SNP-effect mixed linear model (mrMLM) method without Bonferroni correction. Thereafter, many multi-locus GWAS methods have been presented, including the ISIS EM-BLASSO [37], pLARmEB [38], FASTmrEMMA [39], FASTmrMLM [40], and pKWmEB [41] to detect quantitative trait nucleotides (QTNs). In these models, MLM stands for mixed linear model (Q+K model); FAST stands for factored spectrally transformed function; ISIS stands for iterative-modified sure independence screening; EB is expectation–maximization; BLASSO stands for Bayesian least absolute shrinkage and selection operator; EMMAX stands for efficient mixed-model association expedited function; and pKWmEB and pLARmEB stand for Kruskal–Wallis test and LARS algorithm, respectively. An R package called the mrMLM has been developed (https://cran.r-project.org/web/packages/mrMLM/index.html, 1 August 2024), through which these six multi-locus GWAS methods are integrated into the R-based software (V4.0) [42,43]. Because multi-locus GWAS models are relatively closer to true genetic models in plants and animals than existing single-locus GWAS methods, these methods appear to display more robust identification of QTNs with lower false positive rates (FPRs) in the analyses, especially with small-effect quantitative traits [26,36].

It is generally believed that cultivated soybean [*Glycine max* (L.) Merr.] was derived from its wild progenitor, *G. soja* Sieb. et Zucc., approximately 0.8 million years ago in Eastern Asia [44]. The landraces of soybean were subsequently selected from the adaptation of localized *Glycine max* after its domestication [45]. The genetic sources of current elite cultivars in the United States were developed from a small number of landrace accessions [46]. Traditional breeding methods for selection have greatly improved soybean production for economically important traits such as yield, protein, and oil content, but they have not been performed on a large scale to develop cultivars with health-benefiting characteristics. The lunasin gene has been cloned from midmaturation soybean seed [named 2S albumin (*Gm2S-1*)] using a homolog search-based cDNA approach [47], and the bioactivities of lunasin have been assessed in commercial lines in South Korea for lunasin concentrations [48]. Single-nucleotide polymorphisms (SNPs) are the most promising molecular markers in the genome. Because SNPs are the most abundant type of genetic polymorphism and are evolutionarily stable from generation to generation, SNPs represent the ideal form of molecular marker. With the target SNPs identified by multi-locus GWAS, a high-throughput and cost-effective platform will be used to genotype populations and conduct marker-assisted selection (MAS) for genetic studies and trait improvement. The application of MAS in a trait improvement program will be used to shorten breeding cycles and allow a greater genetic gain over time due to more cycles of selection. The genotypic and phenotypic data for the 144 soybean accessions have been collected, and primary data analysis has been conducted. Based on the results, we hypothesize that genetic variants are the key factor in determining lunasin content in soybean seeds. The objective of this study is to disclose the genetic bases of the anticancer bioactive compound lunasin in soybean plants using a multi-locus GWAS with two diverse environments. The information derived from this research should provide valuable information for molecular breeding and marker-assisted selection and contribute to soybean genetic and trait improvement for human health benefits.

## 2. Results

### 2.1. Trait Distribution and Broad Sense Heritability of Assessed Traits

**Figure 1:**
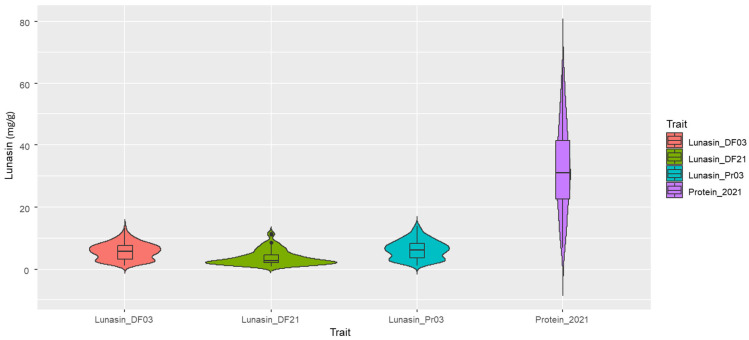
Trait distribution and parametric characters for seed lunasin content in GRIN (2003) and Clayton, NC (2021), in GWAS mapping panel displayed in violin plots. The trait was assessed in defatted and undefatted (protein mg/g) soybean (*Glycine max*) samples in 2003 and lipid-adjusted (defatted) and undefatted (protein) soybean flour.

**Figure 2:**
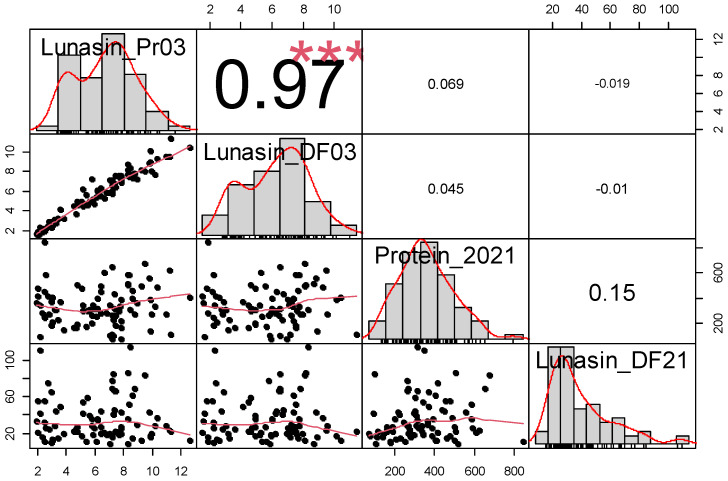
Correlation between lunasin content of defatted and undefatted soybean flour (GRIN, 2003) and lipid-adjusted and undefatted soybean flour (Clayton, NC, 2021). * The significance of correlation (* *p* < 0.05, ** *p* < 0.005, *** *p* < 0.005). The distribution of each trait is displayed in histograms, and correlations between two traits are shown in scatter plots.

The lunasin content in the soybean seeds of the GWAS mapping panel varied significantly across different environments. The frequency distribution of the assessed traits in the GWAS mapping panel was evaluated using the Shapiro–Wilk test for normality (Table 1). Only the lunasin content of undefatted flour in 2021 (Lunasin_Pr21) followed a normal distribution. Negative skewness in the distribution of lunasin content in defatted flour (Lunasin_DF03) and a kurtosis value greater than 3 were observed in the mapping population (Table 1; Figure 1). The trait distribution of Lunasin_Pr21 exhibited significant variation, even after the data were transformed to a 1/10 scale, indicating potential biological or experimental factors influencing the trait’s expression. The coefficient of variation (CV) for each assessed trait showed some consistency, except for Lunasin_DF21. The CV values ranged from 43% to 46% for Lunasin_DF03 and Lunasin_Pr21, whereas a CV of 66.82% was observed for lunasin content in 2021. The mean lunasin content in soybean seeds from these two environments differed significantly, with some extreme outliers identified (Table 1). Estimates of trait correlations showed that the correlogram of lunasin content and protein across different environments varied remarkably (Figure 2). Among the four phenotypes assessed, the correlation coefficient (r) between the two methods of assessed lunasin content was 0.97 (*p* < 0.001) in 2003. However, no significant correlation (r = 0.15) was identified between undefatted flour and lipid-adjusted soybean flour for the lunasin concentration in soybean seeds. The broad-sense heritability (H^2^) of for the trait of seed lunasin content (mg/g of dry, defatted (Lunasin_DF03) and lipid-adjusted (Lunasin_DF21) samples) across the two years was 27%. The Panel–Year interactions played a significant role in the molecular formation of lunasin in soybean seeds in these two markedly different environments, as indicated by our two-way ANOVA assessment, where the σ_GE_^2^ was 244.5. Due to the cost constraints of this student-centered project, independent duplicates with technical replicates were applied in Lunasin_DF21, and we calculated the Sum Sq and Mean Sq to determine σ_G_^2^ and σ_GE_^2^ for lunasin H^2^ using the type I sum of squares (ANOVA (model) function in R.

### 2.2. Multi-Locu GWAS QTN Mapping

**Figure 3:**
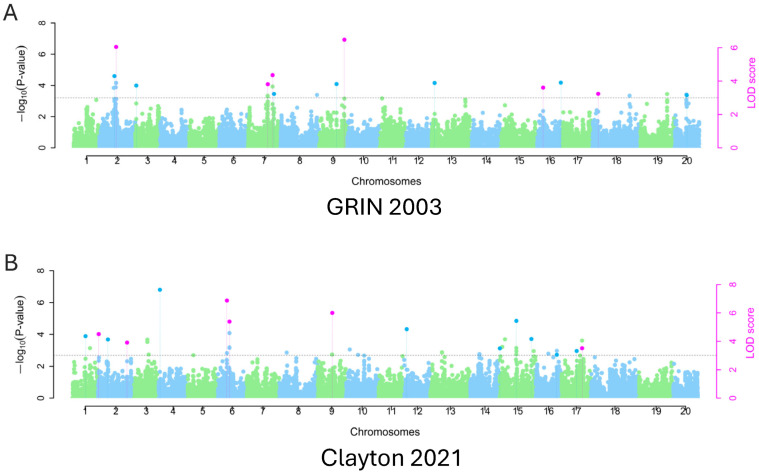
Manhattan plots (showing significant marker–trait associations) for lunasin content in soybean seeds grown in two different environments, GRIN (2003) (**A**) and Clayton, NC (202) (**B**), respectively.

A set of 42,080 SNPs was used in this study, distributed across all 20 soybean chromosomes. A total of 15 chromosomes were detected containing QTNs significantly related to lunasin content in soybean seeds, with LOD scores greater than 3.0, explaining various phenotypic variations identified using the R package mrMLM (v4.0) (Table 2). For significant QTNs, the SNPs were spread out relatively evenly on the chromosomes, and they were most distributed on chromosome 6 and 16 with four SNPs and least distributed on chromosome 4, 5, and 15 with just one SNP. Sixteen significant QTNs were identified in 2003, and 17 QTNs were identified in 2021, shown in the Manhattan plots (Figure 3). Five significant QTNs on chromosomes 3, 13, 16, 18, and 20 were identified by multiple models as being significantly associated with soybean lunasin content based on two years of assessment data. The models ISIS EM-BLASSO, FASTmrMLM, mrMLM, pLARmEB, and FASTmrEMMA detected 13, 12, 12, 12, and 5 significant QTNs, respectively. A total of five chromosomes contained QTNs identified by more than four different models, all with LOD scores greater than 3.0, explaining varying amounts of phenotypic variation over two years of data (Table 2). On chromosome 7, a total of four significant QTNs were detected by different models, mrMLM (three), FASTmrMLM (one), pLARmEB (one), and ISIS EM-BLASSO (two), explaining 3.5% to 11.4% of the phenotypic variation in 2003, with the highest LOD score of 6.6 using the FASTmrMLM model. On chromosome 6, five significant QTNs were identified by the models FASTmrMLM (two), ISIS EM-BLASSO (two), and pLARmEB (two) in 2021, explaining up to 9.2% of the phenotypic variation, with the highest LOD score of 9.5 using the pLARmEB algorithm. Similarly, four significant QTNs were found on chromosome 16 by models ISIS EM-BLASSO (two), pLARmEB (two), FASTmrMLM (one), and mrMLM (one), explaining up to 6.4% of the variation in lunasin content. On chromosome 10, three significant QTNs associated with lunasin content were identified using the models mrMLM (two), pLARmEB (one), and ISIS EM-BLASSO (one), explaining the highest amount of phenotypic variation (30.99%) in 2021 by mrMLM. One significant QTN on chromosome 12 was identified by mrMLM, explaining 26.15% of the second highest phenotypic variation in 2021. On chromosome 2, two significant QTNs associated with lunasin content were identified using the models FASTmrMLM (one), FASTmrEMMA (one), pLARmEB (one), and ISIS EM-BLASSO (one), explaining the highest amount of phenotypic variation (26.1%) in 2003 by FASTmrMLM. A total of two significant QTNs were detected on chromosome 9 by different models, mrMLM, pLARmEB, and ISIS EM-BLASSO, explaining the second highest phenotypic variation in 2003 (16.6%), with the highest LOD score of 6.6 using the FASTmrMLM model. One significant QTN associated with lunasin content was identified on chromosome 15 using the models of FASTmrMLM, pLARmEB, and ISIS EM-BLASSO, with the highest LOD score of 10.2 using the FASTmrMLM algorithm, explaining the high amount of phenotypic variation (17.7%) in 2021 by FASTmrMLM. Additional significant QTNs were identified on chromosomes 3, 4, 5, 13, 14, 18, and 20 using the five different models, respectively (Table 2). The QTN effects for lunasin were relatively diverse in two environments, as listed in Table 2, with an effect of −47 for SNP marker ss715607293 anchored on chromosome 10 and an effect of 32 for SNP marker ss715612259 located on chromosome 12. These two SNPs explained the very high percentage of phenotypic variation, at 31% for ss715607293 with an LOD of 5.2 and 16.6% for ss715612259 with an LOD of 4.7, respectively.

### 2.3. Candidate Genes for Lunasin Content in Soybean Seeds

The candidate genes underlying significant QTNs for lunasin content in soybean seeds were identified using SoyBase JBrowser (soybase.org) Williams 82 (Wm82) genome assembly 6 (https://www.soybase.org/tools/browsers/gbrowse.html?iframe_pathname_suffix=glyma.Wm82.gnm6, 1 August 2024). A total of 28 genes were found near significant QTNs based on the soybean genome browser, using data from two years of field assessments, with 12 candidate genes identified in seeds from 2003 and 15 from 2021. These candidate genes encode pentatricopeptide repeat (PPR) superfamily proteins (nine), transmembrane amino acid transporter proteins (five), ribosomal proteins (three), ribosomal proteins (three), ATP binding (two), RNA binding, the nudix hydrolase 1 (NUDT1) cluster, and others (Table 3). These annotated candidate genes are located close to the regions of significant SNPs. In many cases, more than one candidate gene was found near these SNPs for the trait analyzed, suggesting that the multi-locus GWAS platform has strong detection power for deciphering the genomic structure underlying lunasin content in soybean plants. The pentatricopeptide repeat (PPR) superfamily proteins around SNPs ss715607293 and ss715613090 act as a diverse group of RNA-binding proteins found primarily in plants. They play crucial roles in the regulation of gene expression from the nucleus to organelles such as chloroplasts and mitochondria within various cellular functions, such as RNA processing and editing, RNA stability and protection, translation regulation and RAN maturation, and many other functions in peptide synthesis. These SNPs explained more than 30.9% and 26% of the phenotypic variation in 2021. The expression of ABC transporters (ATP-binding cassette transporters) around the significant SNPs ss715581194 and ss715622529 plays an important role in transporting various substances across cellular membranes. Their key functions include facilitating the movement of a wide range of substrates, including ions, small molecules, peptides, and larger macromolecules, across biological membranes and transporting energy substrates against their concentration gradient, which is critical for maintaining cellular homeostasis and nutrient balance. These significant SNPs explained up to 26% of the phenotypic variation. Moreover, ribosomal proteins (r-proteins) are essential components of ribosomes, the cellular machinery responsible for protein synthesis. Their key functions include contributing to the overall structure and stability of ribosomes and maintaining the integrity of ribosomal architecture. These proteins facilitate cellular function by assisting in the binding of messenger RNA (mRNA) and transfer RNA (tRNA) to the ribosome, and this step ensures the correct assembly of amino acids into peptides following the requirements of the genetic code. These significant SNPs explained various phenotypic variations within two years data (Table 2 and Table 3). NUDT1, or nudix hydrolase 1 or MutT Homolog1 (MTH1), has been paid attention in human research for its enzymatic activity associated with cancer. While it has garnered significant attention in human cancer research, its role in soybean as a gene cluster with high phenotypic variation is also intriguing. A total of 11 *nudix hydrolase 1* genes were identified around a significant SNP (ss715581194) in Glycine max Wm82 genome assembly version 6 (glyma.Wm82.gnm6), and this SNP explained 26.1% of the phenotypic variation. Within these 11 genes, *Glyma.02G130702*, *Glyma.02G131152*, *Glyma.02G131102*, *Glyma.02G131052*, *Glyma.02G131002*, *Glyma.02G130950*, *Glyma.02G130902*, and *Glyma.02G130852* contain 209 nucleotides, while *Glyma.02G130820*, *Glyma.02G130752*, *and Glyma.02G130702* contain 354, 375, and 375 nucleotides. These genes were located on the minus strain of the soybean genome on chromosome 2.

Candidate genes were annotated using SoyBase JBrowser (soybase.org) Williams 82 (Wm82) genome assembly 6 (https://www.soybase.org/tools/browsers/gbrowse.html?iframe_pathname_suffix=glyma.Wm82.gnm6, 1 August 2024).

## 3. Discussion

Lunasin has shown remarkable cancer-preventive, antioxidant, and hypocholesterolemic effects in animal and in vitro trials [5,6,7,8,9,10,11,12]. Overall, tremendous progress has been made in understanding the anticancer bioactive functions of lunasin. However, the genetic basis and inheritance of lunasin have not been fully addressed, and QTL mapping and candidate genes that are associated with lunasin production in soybean have not been defined genetically to the best of our knowledge. More studies are required to elucidate the genetic mechanisms of production for lunasin in soybean seeds to seal the gap between advanced clinical therapeutic success and the lack of genetic information on lunasin inheritance. de Mejia et al. [20] quantified lunasin concentrations in 144 diverse soybean accessions (*Glycine max*) from the USDA Soybean Germplasm Collection (GRIN). Their findings revealed substantial variability in lunasin concentrations among soybean accessions, ranging from 0.1 to 1.3 g per 100 g of flour. The observed differences in lunasin content exceeded 100%, even when soybean lines were cultivated under identical environmental conditions. Similarly, Jeong et al. [8] identified significant phenotypic diversity in lunasin content in Korean soybean accessions, with concentrations ranging from 4.40 to 70.49 mg of lunasin per gram of protein. This pronounced phenotypic variation in lunasin content among soybean seeds highlights its potential as an ideal candidate trait for genome-wide association studies (GWASs) and genetic improvement efforts aimed at enhancing lunasin concentration in soybean plants. Based on these findings, we hypothesize that genetic variants are the primary determinants of lunasin content in soybean seeds. To validate the hypothesis, a total of 251 soybean lines were requested from the GRIN in 2021 and planted in different fields at the Central Crop Research Station of North Carolina State University (Clayton, NC, USA) over the past four years (2021–2024). The lunasin content in seeds harvested in 2021 and 144 lines phenotyped in 2003 were assessed exclusively using the enzyme-linked immunosorbent assay (ELISA) method, primarily due to cost-effectiveness considerations (mainly anti-rabbit polyclonal antibody). The ELISA was selected for its balance of affordability, sensitivity, and accuracy, making it an ideal choice for high-throughput analysis under budgetary constraints. These findings aim to contribute to the understanding of lunasin biosynthesis and its genetic regulation, providing a foundation for future breeding programs to develop soybean varieties with enhanced functional properties.

The multi-locus mrMLM platform aims to select potentially associated markers rather than to identify significant loci or intervals by employing multiple methods to increase the probability of identifying potential significant loci. The models of multi-locus genome-wide association study (GWAS) methodologies embedded in the r package of mrMLM v4.0.2 have been implemented in numerous applications and have demonstrated enhanced uncovering supremacy and improved accuracy in estimating QTN effects compared to previous single-locus GWAS methods [42,49,50]. Rapid multi-locus genome deciphering and scrupulously considering all effects whilst controlling for all genetic backgrounds makes this platform an effective choice for GWAS mapping. Multi-locus GWAS algorithms depend on the random-SNP-effect model with two stages: selecting a reduced number of molecular markers using different algorithms and then deciphering the true associations with multi-locus models, respectively [36]. In the present study, several significant QTNs, including numerous QTNs with small effects, were identified as being associated with lunasin content in soybean seeds, providing valuable insights into the genetic factors influencing this trait (Table 2). These significant QTNs should be used to breed favorable alleles into elite soybean lines via marker-assisted selection (MAS). The genomic regions underlying significant QTNs that explain a substantial portion of the phenotypic variation are likely to contain candidate genes responsible for lunasin production in soybean plants. Notably, these loci include chromosomes 2, 7, 10, 12, and 15, which harbor the genes associated with the target trait. In addition, the identification of small-effect QTNs linked to lunasin content highlights the complexity and dynamic nature of metabolic networks in soybean plants, suggesting that both major and minor loci contribute to the regulation and biosynthesis of this important compound. These findings provide a comprehensive framework for understanding the genetic architecture of lunasin production and offer potential targets for future functional studies and breeding programs. Among the thirty-three QTNs detected within two years of data collection in the present study, nine QTNs explained more than 10% of the phenotypic variation.

Lunasin was identified as a 43-amino acid peptide, presented as a cDNA with an 828 bp transcript (AF005030) in the NCBI (https://www.ncbi.nlm.nih.gov/gene/?term=AF005030, 1 August 2024), which was cloned from mid-maturation soybean seed, encodes a 2S albumin (*Gm2S-1*) based on the previous study [51], is anchored on soybean chromosome 13 (*Glyma.13G154100*), and encodes a bifunctional inhibitor/lipid-transfer protein/seed storage 2S albumin superfamily protein (soybase.org). However, none of the significant QTNs identified in our study were found to be located near this locus. This suggests that the genetic variations associated with the trait may be regulated by different genomic regions or highly impacted by environmental factors, indicating complex genetic architecture that warrants further investigation. Expression of the lunasin gene in mammalian cells resulted in mitotic arrest, ultimately leading to cell death, as demonstrated by Galvez and de Lumen [5], due to its impact on the ability of the kinetochore complex to attach to centromeres during cell mitosis. However, the genetic basis underlying lunasin production is still missing. Based on the multi-locus GWAS in our research, a total of 28 candidate genes near or adjacent to the primary significant QTNs for lunasin content in soybean seeds were identified using SoyBase JBrowser (soybase.org) Williams 82 (Wm82) genome assembly 6 (https://www.soybase.org/tools/browsers/gbrowse.html?iframe_pathname_suffix=glyma.Wm82.gnm6, 1 August 2024). From the genetic analysis of two years of field assessments, 12 candidate genes were identified in the seeds from 2003 and 15 from 2021. The SNPs behind these candidate genes explained more than 26% and 30.9% of the phenotypic variation in 2003 and 2021, respectively. The function of these genes around the significant SNPs plays an important role, which includes facilitating the movement of a wide range of substrates, molecules and energy transport, ribosomal protein (r-protein) components of ribosomes, and the cellular machinery responsible for RNA and protein synthesis. One of the significant SNPs explained 26% of the phenotypic variation on chromosome 2 in 2003 data, defined as a new candidate gene called *NUDT 1* (nudix hydrolase 1). The *NUDT 1* gene has been extensively studied for its enzymatic activity and association with cancer in humans. While it has garnered significant attention in cancer biology, its role in soybean is equally intriguing. In soybean, *NUDT 1* functions as part of a gene cluster characterized by high phenotypic variation, which may have implications for plant development, stress responses, and agricultural productivity. The gene family of nucleoside diphosphate-linked moiety X (*Nudix*) hydrolases composes a large group of genes in living organisms, and it functions to degrade the nucleoside diphosphate-X (NDP-X) to nucleoside monophosphate (NMP) and phosphate-X (P-X) and regulates plant immunity in *Arabidopsis* to initiate pathogen response [52,53]. Plant Nudix hydrolases play very important roles in the plant–pathogen interaction, functioning as important players in the defense mechanisms plants employ against pathogens. The enzymes encoded by this gene family hydrolyze a variety of substrates, including nucleotides and secondary metabolites, are involved in regulating oxidative stress and modulating immune responses, and help plants maintain cellular integrity under pathogen attack, making them crucial components of the plant’s immune system [54]. A total of 11 *NUDT 1* genes were identified around the significant SNP (ss715581194) in *Glycine max* Wm82 genome assembly version 6 (glyma.Wm82.gnm6, 1 August 2024) and this SNP explained 26.1% of the phenotypic variation. These 11 genes contain 209 nucleotides, while three additional family members contain 354, 375, and 375 nucleotides, and these genes were located on a minus strain of the soybean genome on chromosome 2. The expression of these candidate genes is in soybean plants still under investigation.

Heritability is an essential concept in quantitative genetics and a key element in the improvement of genetic attributes through selection. It estimates the proportion of phenotypic variation attributable to genetic factors, providing insight into the selection response and the potential for trait improvement [55]. In plants, the genotype-by-environment interaction plays a significant role in crop production. Hassani et al. [56] studied the location, year, and genotype of both the root and white sugar yield of sugar beet, suggesting that two-way and three-way interactions among these factors affect the stability of root and white sugar yield and impact key traits such as sugar, nitrogen, sodium, and potassium contents among 20 sugar beet genotypes. By using various analytic approaches, their study also identified negative correlations between these key traits and root yield, and the optimal performance of traits and multiple-trait stability were genotype-dependent. The broad-sense heritability of lunasin content in soybean seeds has been estimated at 27%, underscoring the substantial role of genotype–environment interactions in trait expression. This value suggests that lunasin gene expression is strongly influenced by environmental factors, with diverse growing conditions significantly impacting its phenotypic manifestation. Not all high levels of lunasin content are consistent across different environments for soybean lines; while some accessions exhibit elevated content, others display lower levels, highlighting variability influenced by environmental factors. In the soybean seeds of Clayton, North Carolina, a total of 17 significant QTNs were identified by various models for lunasin content, while 15 significant QTNs for lunasin content were found in the seeds of the GRIN from 2003. Some of these QTNs such as on chromosomes 13, 16, and 20 were located adjacent to genomic intervals on the chromosomes; however, none of these QTNs shared the same genomic position, even though they were identified by the same models (Table 2).

The dissection methodology employed in multi-locus GWAS software [42] differs fundamentally from that of traditional single-locus GWASs. In multi-locus GWASs, the analytical framework accounts for the joint effects of multiple loci and often correlates more directly with the specific nucleotide levels where SNPs are anchored. As a result, although the identified QTNs may not be identical across environments or models, they can still exhibit genomic collinearity and collectively contribute to lunasin production through linked or interacting genetic regions. In the mapping panel, we observed several soybean accessions consistently expressing high or low levels of lunasin across diverse environmental conditions. We observed several soybean accessions that consistently expressed either high or low levels of lunasin across these two diverse environmental conditions. This stable expression pattern suggests that these accessions may possess genetically regulated mechanisms influencing lunasin biosynthesis, making them valuable candidates for further genetic analysis and breeding programs. This reinforces the potential functional relevance of the collineated QTNs. While these QTNs may not be universally conserved across models, their co-localization on the genome and consistent phenotypic effects suggest that they can be effectively utilized in marker-assisted selection (MAS). However, careful consideration may be needed in handling these markers, possibly integrating them into multi-locus selection strategies rather than treating them as independent loci. The interaction between genetic and environmental factors complicates breeding efforts aimed at stabilizing lunasin production, as gene expression may fluctuate based on ambient weather conditions such as temperature, rainfall, and soil quality. Studies have demonstrated that genotype–environment interactions can either enhance or suppress the expression of key traits, depending on the environmental context [57]. In the case of soybean, these interactions highlight the necessity of multi-environment trials (METs) to evaluate genotypes across varied climatic zones and to identify stable, high-performing genotypes for lunasin content. While specific studies on lunasin content in soybeans are limited, recent research has demonstrated the effectiveness of multi-environment trials (METs) and genomic tools in evaluating and improving complex traits in soybeans. For instance, a study by [58] identified 22 loci associated with seed weight through a genome-wide association study (GWAS) and estimated the prediction accuracies of genomic selection (GS) and marker-assisted selection (MAS) for this trait. Furthermore, leveraging quantitative trait loci (QTL) mapping and transcriptomic studies has enabled researchers to understand how environmental factors modulate gene expression. Recent studies have utilized transcriptomic analyses to understand how environmental factors influence gene expression in soybeans. For example, research has shown that abiotic stressors, such as drought and temperature extremes, significantly affect biosynthesis pathways. Li et al. [59] studied the physiological indicators of superoxide dismutase (SOD) and peroxidase (POD), which were significantly increased to varying degrees depending on the specific accessions treated with high temperatures. Additionally, the plant hormone abscisic acid (ABA) was elevated, while gibberellin (GA) levels decreased by 2.2-fold in the cotyledon and 1.3-fold in the root. These changes in physiological indicators suggest a complex interaction between environmental factors and gene expression in soybeans, especially regarding the biosynthesis of health-promoting peptides such as lunasin. This highlights the intricate relationship between stress responses and the regulation of key biochemical pathways in the plant. This knowledge underscores the importance of integrating molecular techniques with traditional breeding approaches to enhance trait predictability and stability under varying environmental conditions. The relatively low heritability of lunasin content in soybean seeds, driven by strong genotype–environment interactions, presents a challenge for breeding programs. However, integrating advanced genomic tools and leveraging METs can help breeders identify and develop genotypes that exhibit both high lunasin content and environmental stability, ultimately supporting the production of functional foods with consistent quality.

## 4. Materials and Methods

### 4.1. GWAS Mapping Panel

A total of 251 accessions for multi-locus GWAS analysis including exotic, ancestral, and US modern breeding lines were obtained from the Germplasm Resource Information Network (GRIN) of the U.S. Department of Agriculture Soybean Germplasm Collection (U.S. Department of Agriculture, Agriculture Research Station, University of Illinois, Urbana, IL, USA) in 2003 and 2021. The GWAS mapping panel including exotic accessions, major ancestral lines of U.S. cultivars, and recently released U.S. cultivars was requested from the U.S. Department of Agriculture Soybean Germplasm Collection [(Germplasm Resource Information Network of USDA-ARS (GRIN)] at the University of Illinois Urbana, Champaign, IL, USA which covered all maturity groups and represented diverse genetic origins [20]. These soybean lines were planted, cultivated, and harvested again at the Central Crop Research Station of North Carolina State University for four consecutive years (2021–2024). The Central Crops Research Station of North Carolina State University sits on the western edge of Johnston County (35.66974° N and 78.4926° W) near the city of Clayton, NC. The average precipitation is around 140.5 mm, and the mean daily temperature is 24.5 °C from June to September (National Weather Service, weather.gov). All lines of the GWAS panel were planted in a completely randomized block design (CRBD) with three replications in each environment. A 3 m plot with a row spacing of 65 cm was used, and seeds were planted using a manual push planter at a depth of 2 to 4 cm, with 5 seeds per 30 cm. Standard USDA field and crop management regimes for weed control and fertilizer application were applied across all the replicates. Soybean seeds were sown in May/June; harvesting was conducted in the month of September/October/November depending upon their maturity condition. After harvesting, seeds were stored in brown double-layer paper bags at room temperature inside the seed storage room at FSU and threshed in February next year.

### 4.2. SNP Genotyping and Phenotyping

The leaf samples were collected from young leaves of soybean plants in 1.5 mL microcentrifuge tubes (Fisher Scientific, Pittsburg, PA, USA), and the leaves were stored at −80 °C. DNA was extracted using a DNA extraction kit (Qiagen DNase Plant Extraction, Germantown, MD, USA) in combination with RNase A treatment (Roche Molecular. Biochemicals, Mannheim, Germany). A Picogreen DNA quantitation kit (Invitrogen, Eugene, OR, USA) and BioTek microplate reader (Seattle, WA, USA) with the lamp filter F485 and the emission filter F528 were used to quantify DNA. DNA samples selected for the experiment were normalized to be no less than 200 ng in a 4 µL volume for chip-based genotyping to achieve the optimal concentration and quantity necessary. Because a total of 19,700 accessions, including the wild G. soja and G. max, were genotyped with SoySNP50K high-density SNP arrays (illumine.com) and stored in the USDA Soybean Germplasm Collection [60,61], part of the SNP genotypic data for the mapping panels were retrieved directly from the SoyBase (http://soybase.org/) [62]. However, the lines selected without SNP genotype data were sent to be genotyped using the SNP arrays to obtain the same SNP genotype data for these lines (Molbreeding, Irvine, CA, USA). The genotypes were called by Illumina’s BeadStudio software (Illumina, San Diego, CA, USA, v3.2.23) following the company’s standard protocol. The quality of each SNP was visually inspected using Excel’s sorting function. Non-polymorphic SNPs (minor allele frequency < 5%) were discarded from the dataset manually.

The lunasin content in soybean seeds was only measured from 144 accessions using an enzyme-linked immunosorbent assay (ELISA) method [63,64], with defatted flour (Lunasin_DF03) and undefatted (Lunasin_Pr03) flour requested from the Germplasm Resource Information Network (2003) of the USDA-ARS, and lipid-adjusted soybean flour (Lunasin_DF21) and undefatted flour (Lunasin_Pr21) from crops planted in North Carolina (2021), respectively. For soybean defatting, soybean flour was first passed through a fine sieve (0.5 mm) to ensure uniform particle size. The sieved flour was then mixed with Hexamethylenetetramine (Millipore Sigma, Burlington MA, USA; H11300) at a 1:10 ratio (weight:volume). The mixture was continuously stirred at room temperature for 2 h at 60 °C to facilitate thorough interaction. Afterward, it was filtered to remove excess solvent and left to air-dry overnight under a fume hood to ensure complete solvent evaporation and safe handling of the defatted material. This process yielded defatted soybean flour suitable for downstream applications. Lunasin quantification was carried out by an ELISA and lunasin identification by a Western blot. Several parameters were modified and tested from the original protocol, using the ELISA for lunasin quantification [63] and the Western blot for identification [64]. Several dilutions were tested for the primary lunasin antibody, and these were 1:50, 1:100, 1:200, and 1:400. Several dilutions were also tested for the G. soja protein extracts, and these were 1:100, 1:500, 1:1000, 1:5000, and 1:10,000. We decided on a 1:200 dilution for the primary lunasin antibody and a 1:10,000 dilution for the protein extracts, as these were the optimum parameters for the reaction between the antibody and the antigen. Purified lunasin from *G. max* and commercial synthetic lunasin were tested to build the standard curve (0–1000 ng/mL). Purified lunasin, instead of synthetic lunasin, was used (R^2^ = 0.90–0.95). Quantification of lunasin in extracted soluble protein was performed according to the method described by Cavazos et al. [63] with some modifications conducted to optimize the assay. Briefly, 100 μL of protein extract (diluted 1:10,000) was loaded per well and left to incubate overnight at 4 °C. The well was washed three times with phosphate-buffered saline (PBS, 0.01 M, pH 7.4) before being blocked by 5% bovine serum albumin (BSA, 300 μL per well) for 1 h at 4 °C. The washing process was repeated before incubation with the primary lunasin antibody (rabbit polyclonal) diluted at 1:200 in 3% BSA (50 μL per well) overnight at 4 °C. The wells were washed again, followed by incubation by secondary anti-rabbit IgG conjugated to alkaline phosphatase (1:2000 dilution, 50 μL per well). The washing process was repeated and 100 μL of p-nitrophenyl phosphate (pNPP) was added to each well. The plate was read at 410 nm after 20 min of incubation at room temperature, followed by the addition of 100 μL of NaOH (3 N) to each well to stop the reaction. A reading was taken again at 410 nm after 5 min. Purified lunasin was used to build the standard curve (0–1000 ng/mL). Identification of lunasin by Western blot was performed according to the protocol described in Kusumah et al. [64] with some modifications. Briefly, SDS-PAGE was run to separate proteins in the extract (40 μg in 10 μL solution per well) according to molecular weight before transferring them onto a PVDF membrane. The membrane was then blocked by 5% (*w*/*v*) non-fat milk for 2.5 h at 4 °C, before being washed 6 times by Tris-buffered saline containing 0.1% Tween20 (TBST) and then incubated with primary lunasin antibody (rabbit polyclonal; 1:200 dilution) overnight at 4 °C. The membrane was washed again six times and incubated with secondary anti-rabbit IgG-horseradish peroxidase (1:2000 dilution). The lunasin band was visualized by incubation for 10 min with chemiluminescent reagent, and images were taken using ImageQuant 800 and analyzed by Image.

### 4.3. Multi-Locus GWAS QTN Mapping and Statistical Analysis

The SNP data of 144 soybean accessions assessed by the SoySNP50K SNP arrays were prepared. SNPs from the raw genotype data were filtered with a minor allele frequency (MAF) ≥ 5% and a missing data ratio < 0.1 for association analyses. The population structure of the dataset was analyzed using PCA (principal components analysis) and the program fastSTRUCTURE (http://rajanil.github.io/fastStructure/, 1 August 2024). The mrMLM (multi-locus random-SNP-effect mixed linear model) package was downloaded from http://cran.r-project.org/web/packages/mrMLM/index.html (1 August 2024) following the description in Zhang et al. [42]. Six multi-locus GWAS methods, namely mrMLM [26], FASTmrMLM [40], FASTmrEMMA [39], ISIS_EM-BLASSO [37], pLARmEB [38], and pKWmEB [41], were used to conduct a multi-locus GWAS on 144 diverse accessions with high-quality SNPs. A threshold criterion of an LOD of 3 and above was set to achieve the final set of significant SNPs. To obtain reliable candidate genes for lunasin content, only QTNs with an LOD score ≥ 3 and r^2^ ≥ 4 were searched on the soybean genome in SoyBase (https://soybase.org). In addition, the sequences of both 100 kb up- and down-streams of the defined QTN were investigated as an empirically probable interval of the corresponding QTN. The significant QTNs and associated intervals of chromosomes repeatedly detected in both environments and by a minimum of two methods were defined as dependable QTNs or QTN clusters, respectively. R (www.r-project.org) and its integrated development environment RStudio (posit.co) were used in the multi-locus GWAS, with statistical analyses including agronomic traits, two-way ANOVA, and broad-sense heritability carried out using its native packages. The trait distribution and other parametric characters including means, variance, and standard deviation are displayed in violin plots, using r code within the ggplot2 package (https://r-charts.com/distribution/violin-plot-group-ggplot2/, 1 August 2024). The lunasin content for 2021 was percentage-transferred when it was displayed in the violin plot. The significance level of assessed traits was generated using R package car (type II Wald chi-square tests) (www.r-project.org). The calculation of broad-sense (mean-based) heritability from the ANOVA table of two-way ANOVA was carried out using the equation H^2^ = σ_G_^2^/[σ_G_^2^ + (σ_GE_^2^/e) + (σ_e_^2^/re)], where σ_G_^2^ (variance of genetic factors), σ_GE_^2^ (variance of genotype–environment interactions), and σ_e_^2^ (variance of random effects) were assessed with e (number of environments) and r (number of replicates) normalization [65]. The boxplots were generated to visualize the phenotype distribution among constructed haplotypes using R script (r-project.org). Three biological replicates were included, and statistical significance was analyzed by ANOVA and displayed in an ANOVA table.

## 5. Conclusions

This study provides valuable insights into the genetic basis of lunasin content in soybean seeds, a peptide with notable cancer-preventive, antioxidant, and hypocholesterolemic properties. By analyzing a diverse panel of 144 soybean accessions, we identified 32 significant quantitative trait nucleotides (QTNs) across multiple chromosomes, with key QTNs located on chromosomes 2, 7, 12, and 15. Candidate genes associated with these QTNs were annotated, revealing potential functional pathways involved in lunasin biosynthesis. These findings contribute to a deeper understanding of the genetic factors influencing lunasin production, which could inform future breeding strategies for enhanced health benefits in soybeans.

## Figures and Tables

**Table 1:** Agronomic parameters of assessed traits in GWAS mapping panel in GRIN (2003) and Calyton, NC (2021). Significant level: * < 0.05, **< 0.01, *** < 0.001.

Trait	Mean	Range	CV (%)	SE	Skewness	Kurtosis	W Value (*p* < 0.05)
Lunasin_Pr03	6.32	10.74	43.08	0.32	0.01	2.15	0.96 *
Lunasin_DF03	5.79	9.88	43.03	0.29	−0.08	2.14	0.96 *
Lunasin_Pr21	338.09	772.87	46.16	17.23	0.56	3.25	0.97
Lunasin_DF21	35.29	106.49	66.82	2.6	1.33	4.41	0.87 ***

**Table 2:** QTNs detected for lunasin content in soybean seeds grown in two diverse environments, GRIN (2023) and Clayton, NC (2021), using multi-locus GWAS models.

Trait Name	Method	SNP	Chr.	Marker Position	QTN Effect	LOD Score	‘−log10(P)’	r^2^ (%)	MAF	Genotype
Lunasin_DF03	mrMLM	ss715595316	6	5184562	1.2504	4.0461	4.7999	8.3756	0.1179	G
Lunasin_DF03	mrMLM	ss715596616	7	17043096	0.8142	5.8761	6.7049	6.9945	0.3302	C
Lunasin_DF03	mrMLM	ss715633674	19	3035283	−8.3977	5.5739	6.392	30.7161	0.0047	G
Lunasin_DF03	FASTmrMLM	ss715595316	6	5184562	0.8255	3.9564	4.7058	4.9591	0.1157	G
Lunasin_DF03	FASTmrMLM	ss715595189	6	50535934	−1.1725	3.0827	3.7832	8.7577	0.1065	A
Lunasin_DF03	FASTmrMLM	ss715596616	7	17043096	0.421	3.0624	3.7616	2.5404	0.3241	C
Lunasin_DF03	FASTmrEMMA	ss715579276	1	40092915	1.2741	4.0034	4.7551	5.5541	0.3056	T
Lunasin_DF03	FASTmrEMMA	ss715586961	3	793778	1.3682	3.5775	4.3071	6.8987	0.3704	A
Lunasin_DF03	FASTmrEMMA	ss715596420	7	15552442	−1.1375	3.6375	4.3703	4.0045	0.2639	A
Lunasin_DF03	pLARmEB	ss715580426	1	54004529	0.7868	3.1176	3.8203	3.6523	0.088	G
Lunasin_DF03	pLARmEB	ss715581194	2	13683259	−1.5026	7.0063	7.871	15.4191	0.0926	G
Lunasin_DF03	pLARmEB	ss715590292	5	1942075	0.4423	4.5676	5.3456	3.0459	0.412	A
Lunasin_DF03	pLARmEB	ss715595316	6	5184562	0.8666	5.0072	5.8036	5.4654	0.1157	G
Lunasin_DF03	pLARmEB	ss715596608	7	16974662	0.5261	3.3406	4.0567	3.967	0.3241	G
Lunasin_DF03	pLARmEB	ss715604755	9	47022602	−0.6594	4.5291	5.3054	5.7395	0.2824	T
Lunasin_DF03	pLARmEB	ss715616380	13	42752378	0.3751	3.275	3.9873	2.1377	0.412	T
Lunasin_DF03	pLARmEB	ss715628168	17	7370439	−0.5191	4.9246	5.7177	3.9145	0.3426	A
Lunasin_DF03	ISIS EM-BLASSO	ss715579317	1	41141330	0.535	5.766	6.5909	4.3876	0.3889	G
Lunasin_DF03	ISIS EM-BLASSO	ss715580042	1	50725197	0.6076	5.4564	6.2702	3.7298	0.1898	A
Lunasin_DF03	ISIS EM-BLASSO	ss715580426	1	54004529	0.8918	4.4724	5.2462	4.6926	0.088	G
Lunasin_DF03	ISIS EM-BLASSO	ss715581194	2	13683259	−0.9361	3.1766	3.8829	5.9838	0.0926	G
Lunasin_DF03	ISIS EM-BLASSO	ss715595316	6	5184562	0.606	3.6641	4.3983	2.6723	0.1157	G
Lunasin_DF03	ISIS EM-BLASSO	ss715596608	7	16974662	0.4661	4.3235	5.0905	3.1138	0.3241	G
Lunasin_DF03	ISIS EM-BLASSO	ss715597517	7	37004443	−0.6891	5.3944	6.2058	4.0617	0.1389	G
Lunasin_DF03	ISIS EM-BLASSO	ss715599447	8	13272509	−0.7145	3.3954	4.1147	1.4537	0.0463	A
Lunasin_DF03	ISIS EM-BLASSO	ss715604755	9	47022602	−0.572	4.6519	5.4335	4.3192	0.2824	T
Lunasin_DF03	ISIS EM-BLASSO	ss715628168	17	7370439	−0.4974	4.6701	5.4525	3.5938	0.3426	A
Lunasin_DF21	mrMLM	ss715588993	4	51820047	−15.7929	6.7159	7.5719	7.4369	0.0875	G
Lunasin_DF21	mrMLM	ss715591002	5	34359243	−6.6651	4.0003	4.7519	4.1176	0.4	C
Lunasin_DF21	mrMLM	ss715607293	10	43465671	−47.9847	5.2081	6.0124	30.9931	0.0187	C
Lunasin_DF21	mrMLM	ss715608045	10	49696358	10.6285	6.4119	7.2585	6.1079	0.1562	G
Lunasin_DF21	mrMLM	ss715613090	12	4402508	−24.2842	5.6262	6.4462	26.1584	0.0688	A
Lunasin_DF21	mrMLM	ss715623919	16	28465014	−8.3573	5.1897	5.9933	6.4143	0.4125	A
Lunasin_DF21	mrMLM	ss715629312	18	1735923	6.1196	3.319	4.0339	2.8076	0.2562	A
Lunasin_DF21	mrMLM	ss715638485	20	44108146	7.0304	3.5643	4.2931	2.518	0.15	T
Lunasin_DF21	FASTmrMLM	ss715591002	5	34359243	−4.567	3.6677	4.4021	3.6408	0.4146	A
Lunasin_DF21	FASTmrMLM	ss715592971	6	12980012	−5.8621	3.3236	4.0387	4.2342	0.2073	A
Lunasin_DF21	FASTmrMLM	ss715594520	6	45930385	−5.7135	4.6139	5.3939	5.5865	0.3841	C
Lunasin_DF21	FASTmrMLM	ss715612259	12	33175894	12.4064	6.1152	6.9521	8.6429	0.1159	T
Lunasin_DF21	FASTmrMLM	ss715616757	13	16561515	−4.0125	3.0799	3.7802	2.8878	0.4939	A
Lunasin_DF21	FASTmrMLM	ss715618992	14	44255136	−8.8116	5.3863	6.1974	7.4492	0.1524	C
Lunasin_DF21	FASTmrMLM	ss715622529	15	49657138	−10.779	10.211	11.1535	17.7085	0.2988	C
Lunasin_DF21	FASTmrEMMA	ss715612259	12	33175894	32.5837	4.7315	5.5165	16.6431	0.1159	T
Lunasin_DF21	FASTmrEMMA	ss715622529	15	49657138	−3.37 × 10^−5^	3.6424	4.3755	4.23 × 10^−11^	0.2988	C
Lunasin_DF21	pLARmEB	ss715585714	3	36302681	−7.1031	7.3819	8.2573	3.0745	0.3415	G
Lunasin_DF21	pLARmEB	ss715592971	6	12980012	−10.0753	9.5639	10.4929	4.7987	0.2073	A
Lunasin_DF21	pLARmEB	ss715594507	6	45436808	5.7038	4.8581	5.6484	1.9438	0.3171	T
Lunasin_DF21	pLARmEB	ss715607342	10	44167653	−14.8486	7.1929	8.063	4.1257	0.061	C
Lunasin_DF21	pLARmEB	ss715612259	12	33175894	13.9585	8.9721	9.8878	4.1975	0.1159	T
Lunasin_DF21	pLARmEB	ss715622529	15	49657138	−7.622	6.2133	7.0534	3.3971	0.2988	C
Lunasin_DF21	pLARmEB	ss715624628	16	33733586	−5.4859	3.485	4.2094	1.5313	0.2195	C
Lunasin_DF21	ISIS EM-BLASSO	ss715592975	6	12984798	−8.1046	6.5187	7.3686	9.2736	0.2622	G
Lunasin_DF21	ISIS EM-BLASSO	ss715594520	6	45930385	−4.9599	3.781	4.5214	4.2099	0.3841	C
Lunasin_DF21	ISIS EM-BLASSO	ss715607342	10	44167653	−14.3003	5.7142	6.5373	9.974	0.061	C
Lunasin_DF21	ISIS EM-BLASSO	ss715612259	12	33175894	9.0802	3.6503	4.3838	4.6298	0.1159	T
Lunasin_DF21	ISIS EM-BLASSO	ss715616757	13	16561515	−4.0037	3.1842	3.891	2.8752	0.4939	A
Lunasin_DF21	ISIS EM-BLASSO	ss715622529	15	49657138	−8.1201	6.2863	7.1288	10.0496	0.2988	C
Lunasin_DF21	ISIS EM-BLASSO	ss715624628	16	33733586	−7.8726	5.2596	6.0659	8.2199	0.2195	C

**Table 3:** Candidate genes or gene clusters around the significant QTNs for lunasin content in soybean seeds.

Environment	QTN	Chromosome	Genomic Position	Candidate Gene ID	Reference Genome	Functional Annotation
GRIN, 2003	*qL-01*	2	12034409	Glyma.02G123302	Wm82.gnm6	RAB geranylgeranyl transferase alpha subunit 1
GRIN, 2003	*qL-02*	2	13683259	Glyma.02G131052	Wm82.gnm6	Nudix hydrolase 1 (NUDT1) cluster
GRIN, 2003	*qL-03*	3	793778	Glyma.03G008400	Wm82.gnm6	Peptide chain release factor
GRIN, 2003	*qL-04*	7	17043096	Glyma.07G144500	Wm82.gnm6	mRNA capping enzyme family protein
GRIN, 2003	*qL-05*	7	35777062	Glyma.07G177100	Wm82.gnm6	Pentatricopeptide repeat (PPR-like) superfamily protein
GRIN, 2003	*qL-06*	7	37004443	Glyma.07G179400	Wm82.gnm6	Embryo defective 1273 protein
GRIN, 2003	*qL-07*	9	47022602	Glyma.09G238700	Wm82.gnm6	ZF-HD homeobox protein cluster
GRIN, 2003	*qL-08*	13	13819188	Glyma.13G044000	Wm82.gnm6	ATP-binding ABC transporter
GRIN, 2003	*qL-09*	16	4380977	Glyma.16G047300	Wm82.gnm6	ATP binding/protein serine/threonine kinase
GRIN, 2003	*qL-10*	16	36170992	Glyma.16G179400	Wm82.gnm6	Pentatricopeptide repeat (PPR) superfamily protein
GRIN, 2003	*qL-11*	18	5146121	Glyma.18G059800	Wm82.gnm6	Pentatricopeptide repeat-containing protein
GRIN, 2003	*qL-12*	20	34499736	Glyma.20G083300	Wm82.gnm6	Ribosomal protein S3
Clayton, NC 2021	*qL-01*	3	36302681	Glyma.03G007400	Wm82.gnm6	Transmembrane protein
Clayton, NC 2021	*qL-02*	4	51820047	Glyma.04G214500	Wm82.gnm6	Ribosomal protein L17 family protein
Clayton, NC 2021	*qL-03*	5	34359243	Glyma.05G129200	Wm82.gnm6	Transmembrane protein
Clayton, NC 2021	*qL-04*	6	12980012	Glyma.06G156700	Wm82.gnm6	Transmembrane amino acid transporter family protein
Clayton, NC 2021	*qL-05*	6	45436808–45930385	Glyma.06G253700	Wm82.gnm6	Transmembrane protein 184C-like
Clayton, NC 2021	*qL-06*	10	43465671	Glyma.10G162500	Wm82.gnm6	Pentatricopeptide repeat (PPR) superfamily protein
Clayton, NC 2021	*qL-07*	10	44167653	Glyma.10G168600	Wm82.gnm6	Pentatricopeptide repeat (PPR) superfamily protein
Clayton, NC 2021	*qL-08*	10	49696358	Glyma.10G230100	Wm82.gnm6	Pentatricopeptide repeat (PPR) superfamily protein
Clayton, NC 2021	*qL-09*	12	4402508	Glyma.12G060101	Wm82.gnm6	Pentatricopeptide repeat (PPR) superfamily protein
Clayton, NC 2021	*qL-10*	12	33175894	Glyma.12G162300	Wm82.gnm6	30S ribosomal protein S20
Clayton, NC 2021	*qL-11*	13	16561515	Glyma.13G068200	Wm82.gnm6	Peptide transporter 1
Clayton, NC 2021	*qL-12*	14	44255136			
Clayton, NC 2021	*qL-13*	15	49657138	Glyma.15G245700	Wm82.gnm6	Pentatricopeptide repeat (PPR) superfamily protein
Clayton, NC 2021	*qL-14*	16	28465014	Glyma.16G116600	Wm82.gnm6	Pentatricopeptide repeat (PPR) superfamily protein
Clayton, NC 2021	*qL-15*	16	33733586	Glyma.16G160200	Wm82.gnm6	Transmembrane amino acid transporter family protein
Clayton, NC 2021	*qL-16*	18	1735923	Glyma.18G022400	Wm82.gnm6	Transmembrane amino acid transporter family protein
Clayton, NC 2021	*qL-17*	20	44108146	Glyma.20G170700	Wm82.gnm6	RAN binding protein 1

## Data Availability

The original contributions presented in this study are included in the article.
